# Bridging the expertise gap: how AI-assisted stroke detection levels the playing field in emergency medicine

**DOI:** 10.1007/s00330-025-12260-2

**Published:** 2025-12-19

**Authors:** Florian Raab, Quirin D. Strotzer

**Affiliations:** Department of Radiology, University Hospital Regensburg, Regensburg, Germany

A fundamental question regarding the adoption of artificial intelligence (AI) tools in clinical practice centers on whether AI assistance benefits all medical professionals equally or whether its impact varies according to baseline expertise. This question is particularly relevant in stroke care, where the maxim “time is brain” demands rapid and accurate diagnosis across diverse clinical settings with varying levels of specialized expertise available.

The featured study by Kim et al [1] elegantly addresses this issue through a multi-reader, multi-case crossover study design testing a preclinical deep learning algorithm for acute ischemic stroke detection on MRI. The readers were stratified into three distinct groups: clinicians without radiology training, radiology residents, and board-certified non-neuroradiologists. Hence, this study captures the reality of modern healthcare, where stroke patients may initially be evaluated by professionals with widely varying levels of neuroimaging expertise.

The study’s most significant finding is the differential impact of the deep learning algorithm across reader groups. While clinicians without radiology training showed substantial improvements in diagnostic performance (AUC increased from 0.90 to 0.93 and sensitivity from 0.77 to 0.88 when supported by the AI tool), more experienced readers showed only minimal changes.

This pattern suggests that AI functions as a diagnostic equalizer, bridging expertise gaps, particularly where specialized knowledge is limited—a potentially life-saving benefit in settings with no experienced readers available. The improvement in inter-reader agreement across all groups (from 0.86 to 0.92 for overall acute ischemic stroke detection with AI support) has profound implications for clinical consistency and quality assurance in multi-site healthcare systems. Notably, AI assistance preserved specificity while enhancing sensitivity across all reader groups, addressing concerns about increased false-positive rates that could lead to unnecessary interventions.

These findings have immediate relevance for healthcare system implementation strategies. The data suggest that AI deployment may be most beneficial in settings where initial stroke evaluation is performed by clinicians with limited neuroimaging expertise, precisely the scenarios encountered in many emergency departments, particularly in resource-limited or rural settings [2].

The study’s demonstration that residents achieved the highest baseline performance, despite having less experience than attending radiologists, highlights an important consideration for AI implementation. This counterintuitive finding may reflect the intensity of current training programs and the frequency with which residents encounter stroke cases during their education. It also highlights the need for ongoing training among experienced practitioners and underscores that even experienced readers may benefit from AI assistance when working outside their primary areas of expertise.

The improvement in diagnostic confidence among clinicians represents another crucial finding. Enhanced confidence in diagnostic decision-making can translate into more decisive clinical actions, potentially reducing time to treatment, a critical factor in stroke outcomes [3].

This research article contributes to a growing body of evidence suggesting that AI’s greatest impact may be in augmenting the capabilities of less specialized practitioners rather than replacing expert judgment. This pattern has been consistently observed across various medical imaging applications, from chest radiograph interpretation to breast cancer detection [4–6]. Such AI-assisted diagnostic tools also hold significant promise for primary care settings, where they could enable general practitioners to handle more complex cases independently, thereby reducing referral pressure on specialists and improving overall healthcare system efficiency.

These implications extend beyond stroke care to emergency medicine more broadly. Emergency physicians frequently encounter clinical scenarios requiring rapid interpretation of imaging studies across multiple specialties. Similarly, the looming shortage of specialist physicians, with projections indicating a global shortfall of nearly 400,000 doctors across OECD countries by 2030, makes AI-supported primary care increasingly essential [7]. AI systems that can provide reliable decision support across diverse pathologies could significantly enhance both emergency care quality and primary care capacity, ultimately creating a more resilient healthcare system where first-contact physicians are empowered to manage a broader scope of conditions while ensuring appropriate specialist referrals when needed.

The study by Kim et al provides valuable evidence that AI-assisted stroke detection can serve as a force for diagnostic democratization, improving performance among less experienced readers while maintaining high standards among experts. This pattern suggests that AI implementation strategies should be tailored to institutional needs and levels of reader expertise. Looking forward, the development of AI systems should focus on creating tools that not only achieve high diagnostic accuracy but also provide appropriate decision support calibrated to user expertise levels. Future AI systems might incorporate adaptive interfaces that provide different levels of assistance based on user profiles and confidence levels. Multimodal AI, such as vision-language models, could provide a foundation for more individualized suggestions but still faces significant limitations in diagnostic accuracy and clinical validation [8].

The ultimate goal is not to replace clinical expertise but to ensure that high-quality diagnostic capabilities are available regardless of the specific clinical setting or the background of the initial evaluating physician. In the context of stroke care, where every minute counts, such democratization of diagnostic excellence could translate into improved outcomes for patients across diverse healthcare environments.

Like every study, it does not come without limitations. Its retrospective, single-center, single vendor design, use of a system that is still under development and not openly available, and potential case selection bias constrain generalizability, and the reader group may not reflect the full range of clinical practice. The Likert scale for confidence scoring may also oversimplify how clinicians express uncertainty. Finally, the study focuses on diagnostic accuracy without addressing workflow, resource, ethical, or patient outcome considerations. Even so, it provides a useful starting point for future work to address these broader real-world questions.

Moving toward broader AI use in clinical practice, studies like this provide the evidence base for thoughtful integration that enhances rather than disrupts workflows and ensures that advanced diagnostic tools benefit patients across all care settings. Because successful AI integration does not depend solely on diagnostic performance, future research should address practical issues, including workflow integration, cost-effectiveness, and the long-term impact on clinical outcomes [9].
